# The Relationship Between Atrial Cardiopathy Biomarkers and Prognosis of Patients With Embolic Stroke of Undetermined Source

**DOI:** 10.3389/fcvm.2022.829361

**Published:** 2022-03-11

**Authors:** Yuye Ning, Meng Wei, Wenfeng Song, Guogang Luo

**Affiliations:** Stroke Centre and Department of Neurology, The First Affiliated Hospital of Xi'an Jiaotong University, Xi'an, China

**Keywords:** ESUS, atrial cardiopathy, biomarkers, prognosis, prevention

## Abstract

**Objective:**

To compare the proportion of atrial cardiopathy in patients with embolic stroke of undetermined source (ESUS) and other non-cardiac strokes, and to evaluate the prognostic value of atrial cardiopathy biomarkers in patients with ESUS.

**Methods:**

This retrospective study enrolled patients with ischemic stroke from January 2018 to April 2020 in a single stroke center, and compared the proportion of atrial cardiopathy in (1) ESUS group, (2) large artery atherosclerosis (LAA) group, and (3) small-vessel occlusion (SVO) group. Then, it compared the baseline characteristics between ESUS patients with atrial cardiopathy and cardioembolism (CE) group. In addition, the relationship was compared between the biomarkers of atrial cardiopathy and prognosis in patients with ESUS.

**Results:**

In total, 316 patients with ischemic stroke were included that included 105 (33.23%) ESUS, 84 (26.58%) LAA, 73 (23.10%) SVO, and 54 (17.09%) CE. Among these patients, patients with ESUS were younger, and had lower triglyceride, lower low-density lipoprotein than non-ESUS group. The proportion of atrial cardiopathy in ESUS group was higher than LAA group or SVO group (42.86 vs. 17.86 vs. 8.22%, *p* < 0.001). Compared with non-atrial cardiopathy group, patients with atrial cardiopathy were older, had lower EF value, larger left ventricular diameter, and longer PR interval. Among 105 patients with ESUS, 11 (10.78%) cases died, 32 (31.37%) cases had poor functional outcome (mRS >2). In the multivariable model, the risk factor associated with the death risk of patients with ESUS was N-terminal pro-B-type natriuretic peptide (NT-proBNP) >250 pg/ml [*p* = 0.025, hazard ratio (*HR*) = 4.626, 95% *CI*: 1.212–17.652] after a 1-year follow-up.

**Conclusions:**

Atrial cardiopathy is more common in patients with ESUS, and the characteristics of ESUS patients with atrial cardiopathy are similar to those in patients with CE. NT-proBNP >250 pg/ml is related to the risk of death in patients with ESUS.

## Introduction

Nearly 30% ischemic strokes (ISs) have no identifiable causes even after standard diagnostic evaluation, are defined as cryptogenic stroke (CS) (1). A study suggested that most of patients with CS are diagnosed as embolism (2). Therefore, the new term of stroke subclassification: the embolic stroke of undetermined source (ESUS) has been proposed in 2014 to specify CS (3).

Accumulating data suggested that atrial cardiopathy, a pathophysiological concept to describe the abnormal atrial substrate or functions, such as atrial fibrosis, impaired myocyte function, and chamber dilation (4–8), possibly form embolic nidus even without atrial fibrillation (AF). There is an association between atrial cardiopathy biomarkers and occurrence of stroke, such as increased p-wave terminal force V1 (PTFV1) (9) on the electrocardiography (ECG), left atrial enlargement (10, 11), increased N-terminal pro-B-type natriuretic peptide (NT-proBNP) (12) in serum. Therefore, they assumed that atrial cardiopathy, indicating abnormal atrial structure and function, might increase stroke risk and this happens even before AF occurs.

Besides, in-depth study on the correlation between biomarker and increased stroke risk is needed. There are limited reports to evaluate and expound the proportion of atrial cardiopathy in ESUS compared with non-cardiac stroke. There are even less studies on the association between atrial cardiopathy biomarkers with the prognosis of patients with ESUS. Therefore, we aimed to examine the association between these biomarkers and patients with ESUS.

## Methods

### Study Population

This study enrolled patients with IS retrospectively from January 2018 to April 2020 in Stroke Centre and Department of Neurology, the First Affiliated Hospital of Xi'an Jiaotong University. Patients with the conformation of new stroke onset through diffusion weighted image (DWI) and adequate clinical evaluation as follows were included: brain CT angiography (CTA) and/or magnetic resonance angiography (MRA) and/or digital subtraction angiography (DSA), ultrasound of cervical vessels, 12-lead ECG, echocardiography, dynamic ECG, and NT-proBNP. We excluded patients who have multiple etiologies or have other definite etiologies, such as moyamoya disease, arterial dissection, and arteritis.

According to TOAST classification criteria, patients were divided into large-artery atherosclerosis (LAA) group, small-vessel occlusion (SVO) group, cardioembolism (CE) group, and ESUS group. The diagnosis criteria for ESUS include the following steps: (1) non-lacunar IS by MRI or CT; (2) exclusion of ≥50% luminal stenosis in extracranial or intracranial arteries using vascular imaging; (3) exclusion of cardioembolic causes; and (4) exclusion of other uncommonly determined causes of stroke (arteritis, dissection, migraine, and drug misuse). This research was approved by the Ethics Committee of the First Affiliated Hospital of Xi'an Jiaotong University (XJTU1AF2020LSK-066), Shaanxi, China.

### Clinical Information

Baseline demographic information were abstracted from electronic medical records, such as sex, age, and medical history (hypertension, diabetes, coronary heart disease, hemorrhagic, and IS). The severity of ESUS was defined with the National Institutes of Health Stroke Scale (NIHSS). Brain MRI was reviewed for each patient to examine a degree of white matter hyperintensities based on Fazekas scale. Cardiac evaluation was reviewed for all patients, such as ECG, transthoracic echocardiograms, and 24-h ambulatory ECG monitoring. Left ventricular end diastolic and systolic diameter, left atrial diameter (LAD), and left ejection fraction were obtained from reports. LAD was classified to mild, moderate, and severe, according to standard criteria (13). PFTV1 and PR interval were measured on the first ECG record after admission. PFTV1 was calculated by multiplying the duration (ms) of the terminal negative component of P-wave by its amplitude (μV) in lead V1 on the 12-lead ECG recorded by manual calculation. NT-proBNP was obtained from first electronic records. Atrial cardiopathy was defined as PTFV1 >5,000 μV·ms or NT-proBNP >250 pg/ml or severe left atrial enlargement (women ≥4.7 cm and men ≥5.2 cm) (13, 14).

### Outcome

The incidence of endpoint events within 1 year after discharge. The basic condition and prognosis of patients were followed-up through telephone. In this study, the primary outcome was the mortality rate of patients with ESUS after discharge. Secondary endpoint measures by the modified Rankin Scale (mRS), the score over 2 was defined as poor functional outcome in patients with ESUS. Researcher who called for follow-up and measured this outcome were blinded to the groups of patients.

### Statistical Analysis

Data were expressed as means ± SD or median (interquartile distance) for continuous variable and as percentages for categorical variables performed with. The between-group comparisons of mean were performed using one-way ANOVA. Non-normal data were evaluated by non-parametric Kruskal–Wallis test. Between-group comparisons of categorical variables were evaluated by the χ^2^ or Fisher exact test. Survival analysis chart was performed with GraphPad Prism software version 8.0.1. Kaplan–Meier curve, cox regression, and binary logistic regression were used to investigate the independent risk factor of the poor prognosis of patients with ESUS.

## Results

Cases before 2018 were excluded for the lack of electronic medical records. In total, this study collected 1,220 cases of IS from January 2018 to the April 2020, and 316 cases met the criteria of admission and exclusion, among which 105 cases met the criteria for ESUS diagnosis. According to TOAST classification, LAA 84 cases, SVO 73 cases, and CE 54 cases, LAA and SVO compose of non-ESUS group.

### Baseline Characteristics

The ESUS group was younger than the non-ESUS group (59.35 ± 15.01 vs. 60.71 ± 12.05, *p* = 0.033), and NIHSS score was higher than the non-ESUS group [3.00 (2.00, 8.00) vs. 1.00 (1.00, 4.50), *p* = 0.005]. Compared with the non-ESUS group, the ESUS group had lower hemoglobin content (135.72 ± 2.10 vs. 144.61 ± 1.26, *p* < 0.001), higher D-dimer content [0.88 (0.44, 1.87) vs. 0.50 (0.36, 0.79), *p* < 0.001], lower triglyceride [1.23 (0.87, 1.65) vs. 1.35 (1.00, 1.81), *p* = 0.041], lower low-density lipoprotein (2.20 ± 0.73 vs. 2.49 ± 0.76, *p* = 0.003), and lower total cholesterol content (3.82 ± 0.88 vs. 4.06 ± 0.92, *p* = 0.044). There was no significant difference in gender, other previous medical history, laboratory examination, echocardiography, and ECG parameters between the two groups (Table 1).

**Table 1:** Baseline characteristics comparison between ESUS and non-ESUS groups.

	**ESUS group** **(*n* = 105)**	**Non-ESUS group (*n* = 157)**	***P*-value**
Age, y, mean (SD)	59.35 (15.01)	60.71 (12.05)	0.033
Sex, %, male	65.71	73.25	0.191
Hypertension, %	60.00	59.87	0.984
Diabetes mellitus, %	21.90	31.21	0.098
CAD, %	15.24	9.55	0.163
Prior stroke, %	13.33	16.56	0.477
Smoke, %	42.86	47.77	0.434
NIHSS, M(Q)	3.00 (2.00, 8.00)	2.00 (1.00, 4.50)	0.005
TC, mean (SD)	3.82 (0.88)	4.06 (0.92)	0.044
TG, M (Q)	1.23 (0.87, 1.65)	1.35 (1.00, 1.81)	0.041
HDL, M (Q)	0.96 (0.77, 1.15)	0.95 (0.82, 1.11)	0.899
LDL, mean (SD)	2.20 (0.73)	2.49 (0.76)	0.003
Hb, mean (SD)	135.72 (2.10)	144.61 (1.26)	<0.001
D-dimer, M (Q)	0.88 (0.44, 1.87)	0.50 (0.36, 0.79)	<0.001
EF, M (Q)	68.00 (62.50, 72.00)	69.00 (65.00, 72.00)	0.081
LVDD, M (Q)	49.00 (46.00, 52.00)	48.00 (46.00, 51.00)	0.167
LVDS, M (Q)	30.00 (27.00, 33.00)	29.00 (27.00, 31.00)	0.053
PR interval, M (Q)	158.00 (142.00, 171.00)	160.00 (146.00, 178.00)	0.185
Single APB, M (Q)	16.00 (1.00, 92.5)	17.50 (5.00, 61.00)	0.567
Coupling APB, M (Q)	0.00 (0.00, 5.5)	0.00 (0.00, 3.00)	0.660

*IS, ischemic stroke; ESUS, embolic strokes of undetermined source; CAD, coronary heart disease; NIHSS, the national institutes of health stroke scale; TC, total cholesterol; HDL, high-density lipoprotein cholesterol; Hb, hemoglobin; EF, ejection fraction; LVDD, left ventricular end diastolic diameter; LVDS, left ventricular end diastolic systolic diameter; APB, atrial premature beat*.

**Figure 1:**
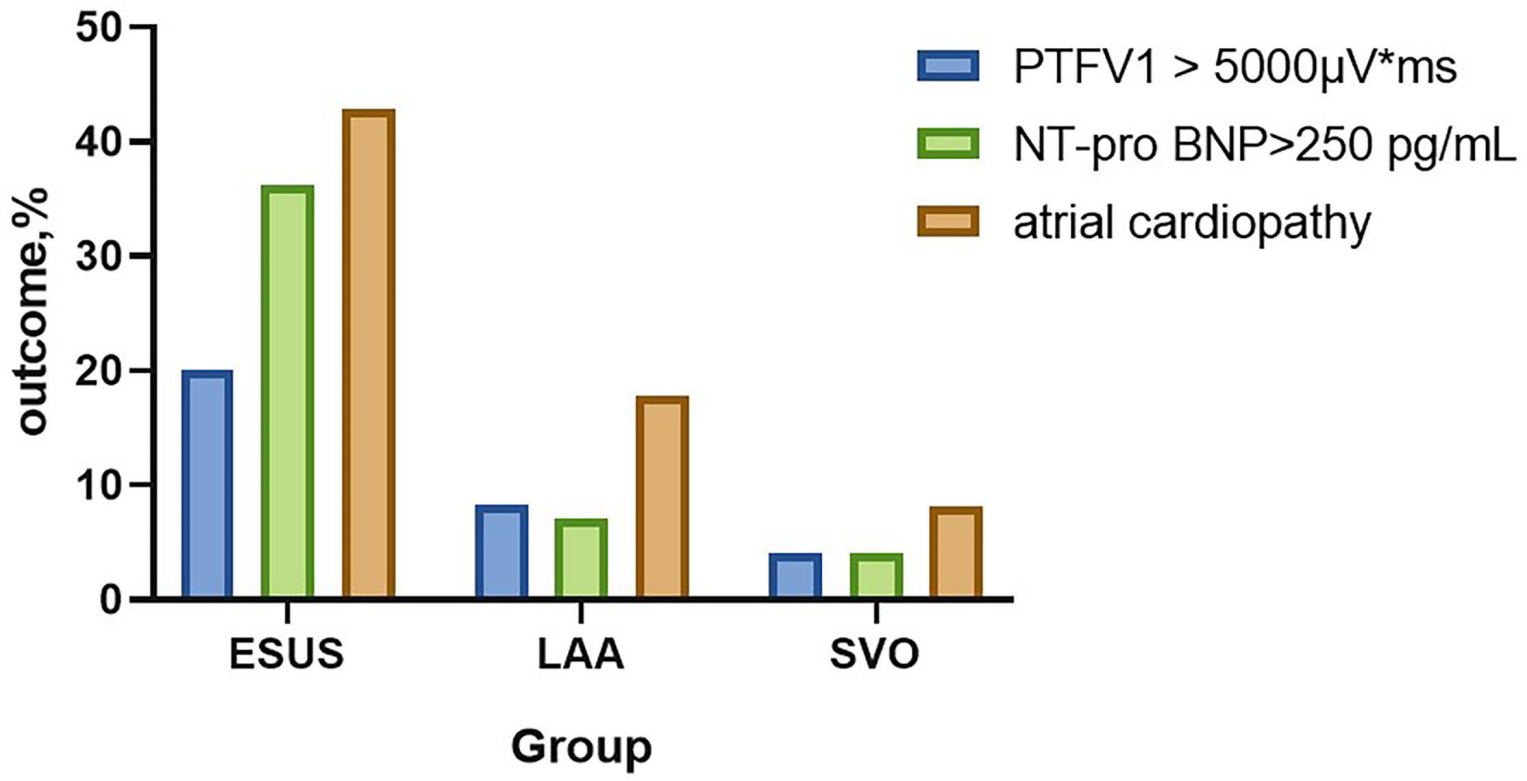
The prevalence of atrial cardiopathy in ESUS/LAA/SVO. ESUS, embolic strokes of undetermined source; LAA, large-artery atherosclerosis; SVO, small-vessel occlusion; PTFV1, P wave terminal force in lead V1; NT-proBNP, N-Terminal pro-brain natriuretic peptide.

Atrial cardiopathy accounted for 42.86% in ESUS, which was more common than the LAA group or SVO group (42.86 vs. 17.86 vs. 8.22%, *p* < 0.001). Although three markers were selected as the diagnostic criteria of atrial cardiopathy, no one in three groups met the criteria of severe left atrial enlargement established by American Ultrasound Association (Table 2; Figure 1) (13).

**Table 2:** The prevalence of atrial cardiopathy in ESUS/LAA/SVO.

	**ESUS (*n* = 105)**	**LAA (*n* = 84)**	**SVO (*n* = 73)**	***P*-value**
PTFV1 >5,000 μV · ms, % (*n*)	20.00 (21)	8.30 (7)	4.11 (3)	0.003
NT-proBNP >250 pg/mL, % (*n*)	36.19 (38)	7.14 (6)	4.11 (3)	<0.001
Atrial cardiopathy, % (*n*)	42.86 (45)	17.86 (15)	8.22 (6)	<0.001

*ESUS, embolic strokes of undetermined source; LAA, large-artery atherosclerosis; SVO, small-vessel occlusion; PTFV1, P wave terminal force in lead V1; NT-proBNP, N-Terminal pro-brain natriuretic peptide*.

We compared the baseline characteristics of ESUS patients with and without atrial cardiopathy. Results showed that ESUS patients with atrial cardiopathy are older (65.28 ± 13.17 vs. 56.00 ± 14.54, *p* = 0.001), have more hypertension (71.11 vs. 1.67%, *p* =0.044), have lower hemoglobin (127.72 ± 21.27 vs. 141.94 ± 19.65, *p* = 0.001), and higher D-dimer [1.02 (0.59, 2.30) vs. 0.70 (0.39, 1.33), *p* = 0.015], but also had lower ejection fraction [66.00 (60.50, 70.00) vs. 69.00 (65.00, 42.00), *p* = 0.026] and larger left ventricular end diastolic (50.16 ± 5.48 vs. 48.11 ± 3.49, *p* = 0.004) or systolic diameter [31.00 (29.00, 34.50) vs. 30.00 (27.00, 32.00), *p* = 0.008], in addition, had longer PR interval (165.24 ± 30.95 vs. 155.93 ± 19.33, *p* = 0.009), more single [65.00 (5.50, 520.5) vs. 9.50 (0.00, 40.25), *p* = 0.001], and paired atrial premature beats [2.00 (0.00, 13.50) vs. 0.00 (0.00, 2.75), *p* = 0.011] and atrial tachycardia [0.00 (0.00, 3.50) vs. 0.00 (0.00, 1.00), *p* = 0.013]. The baseline risk factors, such as diabetes, hypertension, prior stroke, coronary artery disease, and smoking, were similar between two groups (Table 3).

**Table 3:** Baseline characteristics comparison between atrial cardiopathy and no atrial cardiopathy in patients with ESUS.

	**Atrial cardiopathy** **(*n* = 45)**	**No atrial cardiopathy** **(*n* = 60)**	***P*-value**
Age, y, mean (SD)	65.28 ± 13.17	56.00 ± 14.54	0.001
Sex, %, male	71.11	61.67	0.313
Hypertension, %	71.11	51.67	0.044
Diabetes mellitus, %	26.67	18.33	0.307
CAD, %	22.22	10.00	0.085
Prior stroke, %	17.78	10.00	0.246
Smoke, %	46.67	40.00	0.495
NIHSS, M(Q)	4.00 (1.00, 8.50)	3.00 (2.00, 8.00)	0.995
Fazekas, M(Q)	1.00 (1.00, 3.00)	1.00 (0.00, 3.00)	0.562
TC, mean (SD)	3.67 ± 0.90	3.89 ± 0.82	0.120
TG, M (Q)	1.09 (0.86, 1.39)	1.34 (0.87, 1.76)	0.100
HDL, M (Q)	1.02 (0.87, 1.27)	0.91 (0.75, 1.11)	0.157
LDL, mean (SD)	2.05 ± 0.79	2.24 ± 2.22	0.083
Hb, mean (SD)	127.72 ± 21.27	141.94 ± 19.65	0.001
D-dimer, M (Q)	1.02 (0.59, 2.30)	0.70 (0.39, 1.33)	0.015
EF, M (Q)	66.00 (60.50, 70.00)	69.00 (65.00, 42.00)	0.026
LVDD, mean (SD)	50.16 ± 5.48	48.11 ± 3.49	0.004
LVDS, M (Q)	31.00 (29.00, 34.50)	30.00 (27.00,32.00)	0.008
PR interval, mean (SD)	165.24 ± 30.95	155.93 ± 19.33	0.009
Single APB, M (Q)	65.00 (5.50, 520.5)	9.50 (0.00, 40.25)	0.001
Coupling APB, M (Q)	2.00 (0.00, 13.50)	0.00 (0.00, 2.75)	0.011
Atrial tachycardia, M (Q)	0.00 (0.00, 3.50)	0.00 (0.00, 1.00)	0.013

*IS, ischemic stroke; ESUS, embolic strokes of undetermined source; CAD, coronary heart disease; NIHSS, the national institutes of health stroke scale; TC, total cholesterol; HDL, high-density lipoprotein cholesterol; Hb, hemoglobin; EF, ejection fraction; LVDD, left ventricular end diastolic diameter; LVDS, left ventricular end diastolic systolic diameter; APB, atrial premature beat*.

Patients with atrial cardiopathy in ESUS had more hypertension (71.11 vs. 48.15, *p* = 0.021), lower hemoglobin (127.72 ± 21.27 vs. 137.35 ± 15.99, *p* = 0.017) than those in the CE group. The baseline risk factors between the two groups were basically similar (Table 4).

**Table 4:** Baseline characteristics comparison between atrial cardiopathy and cardioembolism (CE) group.

	**Atrial cardiopathy** **(*n* = 45)**	**CE group** **(*n* = 54)**	***P*-value**
Age, y, mean (SD)	65.28 ± 13.17	65.76 ± 16.05	0.868
Sex, %, male	32/13	37/17	0.780
Hypertension, %	71.11	48.15	0.021
Diabetes mellitus, %	26.67	20.37	0.460
CAD, %	22.22	24.07	0.828
Prior stroke, %	17.78	14.81	0.690
Smoke, %	46.67	35.19	0.246
NIHSS, M (Q)	4.00 (1.00, 8.50)	4.00 (2.00, 12.5)	0.251
TC, M (Q)	3.79 (3.02, 4.47)	3.33 (3.03, 4.18)	0.276
TG, M (Q)	1.09 (0.86, 1.39)	1.04 (0.76, 1.61)	0.936
HDL, mean (SD)	1.06 ± 0.28	0.98 ± 0.28	0.494
LDL, M (Q)	2.01 (1.58, 2.56)	1.93 (1.52, 6.40)	0.759
Hb, mean (SD)	127.72 ± 21.27	137.35 ± 15.99	0.017
D-dimer, M (Q)	1.02 (0.59, 2.30)	0.98 (0.76, 1.61)	0.424
EF, M (Q)	66.00 (60.50, 70.00)	65.00 (57.75, 69.25)	0.545
LVDD, M (Q)	50.00 (46.50, 53.00)	48.00 (44.75, 52.00)	0.169
LVDS, M (Q)	31.00 (29.00, 34.50)	31.00 (27.00, 35.25)	0.671
PR interval, M (Q)	162.00 (141.00, 184.00)	173.00 (143.50, 220.5)	0.142

*IS, ischemic stroke; ESUS, embolic strokes of undetermined source; CAD, coronary heart disease; NIHSS, the national institutes of health stroke scale; TC, total cholesterol; HDL, high-density lipoprotein cholesterol; Hb, hemoglobin; EF, ejection fraction; LVDD, left ventricular end diastolic diameter; LVDS, left ventricular end diastolic systolic diameter*.

### Atrial Cardiopathy Biomarkers and Prognosis

Among 105 patients with ESUS, 3 patients were lost to follow-up and 11 patients died accounting for 10.8% (11/102), and there are 32 patients with poor functional outcome (mRS >2) accounting for 31.4% of patients with ESUS after follow-up.

**Figure 2:**
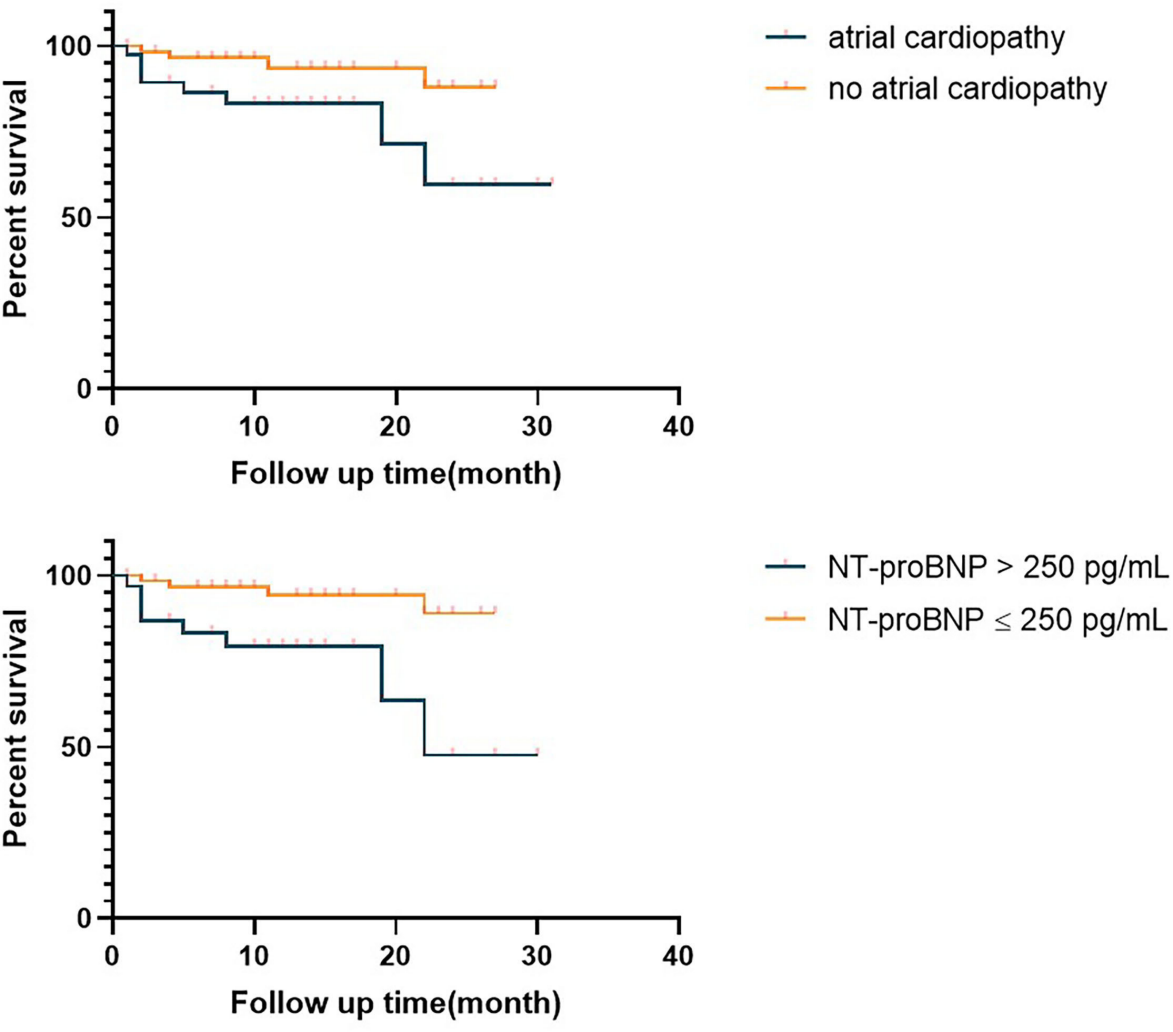
Survival curve of the embolic strokes of undetermined source (ESUS) with atrial cardiopathy and ESUS patients without atrial cardiopathy. NT-proBNP, N-Terminal pro-brain natriuretic peptide.

We compared the prognosis of ESUS patients with atrial cardiopathy and patients without cardiopathy and evaluate the various risk factors. In the Kaplan–Meier analysis, significant statistical differences were found in different group and NT-proBNP >250 pg/ml. These results showed that the risk of death of ESUS patients with atrial cardiopathy was higher than ESUS patients without atrial cardiopathy (Figure 2). The death risk of ESUS patients with NT-proBNP >250 pg/ml was higher than ESUS patients without NT-proBNP >250 pg/ml (*p* = 0.004) (Figure 2). However, age, prior medical history, whether PTFV1 >5,000 μV·ms have no concern with the death risk of death in patients with ESUS (Table 5). Cox regression that included variables significant, the risk factor associated with death was NT-proBNP >250 pg/ml (*p* = 0.025, *HR* = 4.626, 95% *CI*: 1.212–17.652). PTFV1 >5,000 μV·ms, age, sex, and prior medical history were not associated with the death risk (Table 6). In addition, there is no significant correlation between these risk factors above and total poor functional outcome (mRS >2), such as age, sex, past medical history, and two markers in binary logistic regression analysis (Table 7).

**Table 5:** Significant statistical differences were found in Kaplan–Meier analysis.

	**Chi-square**	***P*-value**
Age >60	3.076	0.079
Sex	0.001	0.978
Hypertension	2.393	0.122
Diabetes mellitus	2.213	0.137
CAD	0.681	0.409
Smoke, %	0.521	0.470
NT-proBNP >250 pg/mL	4.835	0.004
PTFV1 >5,000 μV·ms	0.443	0.505
Atrial cardiopathy group	4.477	0.034

*CAD, coronary heart disease; NT-pro BNP, N-terminal pro-B-type natriuretic peptide; PTFV1, p-wave terminal force V1*.

**Table 6:** Significant statistical differences were found in the Cox regression model.

	***P*-value**	**HR**	**Confidence interval**
Age	0.166	1.042	(0.983, 1.105)
Sex (male)	0.560	1.644	(0.309, 8.742)
Hypertension	0.786	1.255	(0.244, 6.462)
Diabetes mellitus	0.111	3.038	(0.774, 11.930)
CAD	0.324	2.158	(0.467, 9.969)
Smoking	0.796	0.816	(0.175, 3.809)
NT-proBNP >250 pg/mL	0.025	4.626	(1.212, 17.652)
PTFV1 >5,000 μV·ms	0.650	0.716	(0.170, 3.023)

*CAD, coronary heart disease; NT-pro BNP, N-terminal pro-B-type natriuretic peptide; PTFV1, p-wave terminal force V1*.

**Table 7:** Significant statistical differences were found in binary logistic regression analysis.

	***P-*value**	**HR**	**Confidence interval**
Age	0.069	1.035	(0.997, 1.075)
Sex (male)	0.871	0.908	(0.281, 2.936)
Hypertension	0.943	0.961	(0.326, 2.829)
Diabetes mellitus	0.491	1.495	(0.476, 4.693)
CAD	0.918	0.932	(0.247, 3.521)
Smoking	0.089	0.354	(0.107, 1.172)
NT-proBNP >250 pg/mL	0.122	2.238	(0.807, 6.207)
PTFV1 >5,000 μV·ms	0.418	0.597	(0.171, 2.080)

*CAD, coronary heart disease; NT-pro BNP, N-terminal pro-B-type natriuretic peptide; PTFV1, p-wave terminal force V1; mRS, the modified Rankin Scale*.

## Discussion

The key to prevent IS to identify its etiology. TOAST classification has been widely used in clinical practice, and it is helpful for the treatment of subtypes, however, this general classification yielded difficulties in the secondary prevention of CS. The definition of CS includes patients with multiple potential causes and patients who have not been completely evaluated. For a long time, because of the complex causes of CS, to formulate their secondary prevention and treatment strategies is very challenging for clinicians. Although the diagnostic criteria of ESUS are more specific than CS, many examinations are not routine clinical examinations (such as, 24-h ECG), so the proportion of patients with ESUS in different countries is quite different. The limitation of screening methods in studies may lead to a relatively wide proportion of patients with ESUS, and about 17% of IS patients meet the ESUS standard (15). However, this may still underestimate the real world proportion, because of the high demand of diagnostic standard and examinations. Eventually a certain number of patients have not been evaluated completely in many studies to date.

In this study, patients with IS were required to have a comprehensive assessment, that was, all patients with IS were required to have the necessary examination results for diagnosing ESUS, only 25.82% of patients with IS had made a comprehensive assessment among 1,220 IS patients with new cerebral infarction focus. Patients with ESUS accounted for 8.6% in patients with ischemic stroke, far less than the reported frequency averaging 17% (15). Note that there are larger number of ischemic stroke patients with incomplete diagnostic evaluation, especially ECG Holter. On the whole, this study showed that there were some problems in patients with IS, such as insufficient etiological assessment and meticulous classification. In particular, only about one-fourth patients with IS had dynamic ECG, which can effectively decrease the detection rate of occult AF.

Left atrial enlargement was associated with stroke recurrence (16, 17), but left atrial size is often measured from echocardiography. However, it cannot fully represent the 3-dimensional size of left atria. Left atrial volume index (LAVi) was more associated with the risk of cardiovascular events (11), especially in patients with ESUS (18). In addition, the research showed that increased LAVi was associated with increased ESUS probability (19). LAVi may be a better biomarker of atrial cardiopathy. However, most patients did not have complete data of height and weight. Therefore, this study measured left atria by 2-dimensional transthoracic echocardiography without index. Disappointingly, none of patients with ESUS met the criteria of severe LAE given by the American Society of Echocardiography. Previous studies had shown that about 63% of patients with CS had atrial cardiopathy (PTFV1 >5,000 μV·ms, or NT-proBNP >250 pg/ml, or severe LAE) (20), or 45% of patients with ESUS had atrial cardiopathy (defined as: female left atrium >38 mm, male left atrium >40 mm, or supraventricular premature beats) (21), or atrial cardiopathy in patients with ESUS accounted for 26.6% (22) (defined as: PTFV1 >5,000 μV·ms, or severe LAE). This study showed that the proportion of atrial cardiopathy in ESUS was 42.86%, which was higher than other non-CE IS (42.86 vs. 17.86 vs. 8.22%). NT-proBNP >250 pg/ml was the most common marker of atrial cardiopathy in the ESUS group. Under different standards, all studies showed that atrial cardiopathy accounted for a certain proportion of patients with ESUS, which was higher than other non-CE IS, and it also confirmed that atrial cardiopathy was one of the potential pathogenesis of ESUS. It could be seen that ESUS patients with atrial cardiopathy had clinical characteristics similar to those of CE, and were different from those of patients without atrial cardiopathy, which confirmed that these patients may have atrial substrate dysfunction. Except for the above three markers, there was no significant difference in PR interval between ESUS and non-ESUS groups, with only 4 cases of paroxysmal supraventricular tachycardia. Since other markers were not routinely examined, the results of most patients were missing (such as, troponin T and cardiac MRI), and relatively accurate results could not be obtained.

Embolic stroke of undetermined source patients with atrial cardiopathy have a higher risk of death compared to ESUS patients without atrial cardiopathy. Among two biomarkers of atrial cardiopathy, NT-proBNP >250 pg/ml associated with the death risk of patients compared with PTFV1 >5,000 μV·ms. Note that no one have severe left atrial enlargement in this study. Therefore, this study only focused on two biomarkers (NT-proBNP and PTFV1). BNP is mainly synthesized and secreted from ventricular myocytes and plays an important role to regulate volume and pressure. Under abnormal conditions, BNP can transform to proBNP which can be split into NT-proBNP, widely used in the clinical diagnosis and prognostic assessment of heart failure. This biomarker is also a signal for adverse cardiac events and non-cardiovascular causes mortality. Multiple evidence suggested that NT-proBNP had prognostic values in patients with cardiovascular diseases (23) and it was the strongest predictor for sudden cardiac death (24). Furthermore, NT-proBNP was independently associated with stroke and AF occurrence (25), and was considered as a biomarker of atrial cardiopathy (8). It may be associated with the prognosis of patients with ESUS even slightly higher than the normal value. In addition, the rise of left ventricular filling pressure can rise the left atrial pressure, then the left atrial myocardium is stretched resulting in increased PTFV1. PTFV1 is a biomarker of atrial enlargement, associated with atrial fibrosis, filling pressure rises, and atrial volume rises. It is suggested that PTFV1 is associated with stroke and AF occurrence (9, 26). A recent study found an association between increased PTFV1 with ESUS (27). Another research showed that increased PTFV1 was associated with occult AF detection but not with recurrent IS (28). This result may be influenced by the fact that all detected patients with AF accepted anticoagulation therapy. The prospective studies with large sample size focused on the association between increased PTFV1 and recurrent IS are needed. Atrial cardiopathy was defined by PTFV1 >5,000 μV·ms or NT-proBNP >250 pg/ml or severe left atrial enlargement based on the association of these biomarkers and stroke risk. Recently, PTFV1 was considered as a more reliable marker associated with stroke (20). In our study, in comparison with PTFV1, NT-proBNP is more relative to the death risk of patients with ESUS. In addition, there were multiple atrial cardiopathy biomarkers, such as prolongation of the PR interval (29), prolonged P-wave durations (30), paroxysmal supraventricular tachycardia (PSVT) (31), and cardiac troponin (cTnT) (25), may be associated with the prognosis of ESUS patients, and more studies are needed to verify their relationship.

The secondary prophylaxis of ESUS is still unclear. Given that lesions mostly originate from embolism in patients with ESUS who may be beneficial from anticoagulation therapy rather than antiplatelet therapy. However, neither NAVIGATE ESUS trial (32) nor RE-SPECT ESUS test (33) can prove that anticoagulant therapy is superior to antiplatelet therapy for patients with ESUS. On the one hand, there are a number of underlying causes associated with ESUS, such as subclinical AF, atrial cardiopathy, unrecognized myocardial infraction, patent foramen ovale, and non-stenosing large-artery atherosclerosis (34). In addition, there is an overlap of these different embolic sources, each patient has an average of 2 embolic sources (21). Therefore, it may affect the clinical trial of secondary prevention for patients with ESUS. Rivaroxban can reduce the risk of IS recurrence in ESUS patients with moderate or severe left atrial enlargement in the NAVIGATE ESUS secondary analysis (35). The reason for the failure of these two trials may be mainly related to the complicated potential mechanism of ESUS, the benefits of anticoagulation therapy may be offset by the causes which may not be suitable for anticoagulation therapy. Given that the ESUS term have great heterogeneity, the prevention strategy should base on potential stroke mechanism. Anticoagulation therapy may be suitable for ESUS with atrial cardiopathy that may be an important subunit in the future study (34, 36). Looking forward to the result of ARCADIA clinical trial which is going to test whether oral apixaban is superior to aspirin in secondary prevention of ESUS patients with atrial cardiopathy (14).

This study was a single-center retrospective study with information bias and introduction selection bias. Since most of the included IS had not been fully evaluated, a large number of patients are excluded from the study, and the accurate proportion of atrial cardiopathy in each non-CE IS cannot be obtained. The patients included in the study did not receive long-term cardiac rhythm monitoring to detect the presence of subclinical AF, some undiscovered AF may be included in the ESUS group, and the patients did not receive further extended cardiac rhythm monitoring (such as, implantable cardiac monitoring devices) after discharge. Compared with transthoracic echocardiography, transesophageal echocardiography may be more accurate in identifying whether there was cardiac disease, further changing the diagnosis. The small sample size increased the possibility of the second error, which may affect the result and lead to the failure to obtain the real result. Furthermore, PTFV1 was calculated by clinicians artificially, and there was a certain error. The LAD was only based on the echocardiography results and should be standardized according to the height and weight of patients. However, most patients lacked the weight data. In addition, there were other biomarkers that were not compared (such as, atrial premature beat, paroxysmal supraventricular tachycardia, and troponin T).

## Conclusion

Since there were few patients with IS who had completed etiological evaluation, the classification was not detailed. Clinicians should strengthen the screening for the potential causes of patients with IS, and especially for occult AF, long-term dynamic ECG should be popularized to improve the detection rate of AF. The proportion of atrial cardiopathy in ESUS was significantly higher than that of LAA and SVO, and NT-proBNP >250 pg/ml was associated with a risk of death in patients with ESUS after discharge, while PTFV1 >5,000 μV·ms was not associated with prognosis, and no patient met the criteria for severe left atrial enlargement.

## Data Availability Statement

The raw data supporting the conclusions of this article will be made available by the authors, without undue reservation.

## Ethics Statement

Written informed consent was obtained from the individual(s) for the publication of any potentially identifiable images or data included in this article.

## Author Contributions

YN and MW drafted the manuscript, illustrated, and captioned all figures. YN, MW, WS, and GL provided critical review, revision of the manuscript, and approved the final draft for publication. All authors contributed to the article and approved the submitted version.

## Conflict of Interest

The authors declare that the research was conducted in the absence of any commercial or financial relationships that could be construed as a potential conflict of interest.

## Publisher's Note

All claims expressed in this article are solely those of the authors and do not necessarily represent those of their affiliated organizations, or those of the publisher, the editors and the reviewers. Any product that may be evaluated in this article, or claim that may be made by its manufacturer, is not guaranteed or endorsed by the publisher.
